# A Summary of the L.G.B. Circular

**Published:** 1918-08-31

**Authors:** 


### MATERNITY AND CHILD WELFARE ACT.

### A Summary of the L.G.B. Circular.

THE following is a brief summary of the important circular issued by Mr. Hayes Fisher under date of August 9, 1918 :—

A Council exercising powers under the Act must appoint a Maternity and Child Welfare Committee. Subject to two-thirds of the committee being members of the Council, persons specially qualified by training or experience in subjects relating to health and maternity may be appointed as members of the committee. Working women should be represented on the committee.

The additional services for which the grant is now available, subject to the Board approving the arrangements, are, chiefly :—

1. Hospital treatment for children up to five years of age. This is not available in cases of ordinary infectious diseases, except ophthalmia neonatorum, but the Board will give a grant towards the provision of beds for epidemic diarrhœa.
2. Lying-in homes for abnormal confinements and for those where the home conditions are unsatisfactory.
3. Home helps. These should be trained at maternity centres and day nurseries, and should be able to do the ordinary domestic duties usually undertaken by the mother.
4. The provision of food for expectant and nursing mothers and for children under five years of age.
5. *Crèches* and day nurseries.
6. Convalescent homes for both mothers and children.
7. Homes for the children of widowed and deserted mothers and for illegitimate children. Provision to be made for the mothers and children to be kept together in certain cases.
8. Experimental work for the health of expectant and nursing mothers and of infants and children under five years of age.

In all cases where accommodation, food, or service is given, a general scale of charges is to be drawn up, but these may be reduced or remitted in individual cases when the circumstances justify this course.

As regards administrative arrangements, the Board thinks it will generally be found desirable to develop a county organisation, but in all cases the county work should be intimately related with that of the local sanitary authority; and, on the other hand, any work separately undertaken by a sanitary authority should be co-ordinated with the county scheme.

Each midwife should be seen by the Inspector of Midwives at least once a quarter. The inspector should, if possible, be a qualified medical woman; if this cannot be secured, she should be a certified and experienced midwife. The supervising authority should secure co-operation between the midwives and the local organisation for maternity and child-welfare work. The Board considers that a competent trained midwife, devoting her whole time to the work, should be able to secure at the present time an income of from £120 to £150 per annum. The Board trusts that all local authorities carrying out maternity and child-welfare schemes will arrange to pay the fees of doctors when called in by midwives during the period of confinement. The midwives should be furnished with a list of doctors willing to attend.

A health visitor should be appointed for each district in which about 400 births take place a year. It is not practicable at the present time to prescribe a special course of training or a standard examination. The qualifications should be : (1) A medical degree, or (2) the full training of a nurse, or (3) the certificate of the Central Midwives Board, or (4) some training in nursing and the health visitor's certificate of a society approved by the Board, or (5) the previous discharge of duties of a similar character in the service of a local authority.

The salary of a whole-time officer acting as health visitor should, as a rule, be not less than £120 a year. The health visitor and the infant protection visitor should, where practicable, be the same person.

The grant is now available for the home nursing of expectant mothers, of lying-in women, puerperal fever, and of measles, whooping-cough, and epidemic diarrhœa in young children and of ophthalmia neonatorum. The local authority may appoint nurses for these purposes or may contract with a district nursing association.

There should be a centre for each health visitor's district. It should be supervised by at least one trained and salaried worker, but unpaid workers interested in its objects should be welcomed. Mothers should be urged to bring their children, whether ailing or not, for advice and for periodical weighings. Every effort should be made to secure the attendance of a doctor—if not at every meeting, at any rate not less often than once a fortnight. Cases found to need anything beyond minor treatment should be referred to their own doctor or to a hospital. A dental clinic should be available for expectant and nursing mothers and for children under five. The Board will sanction the provision of cots at a centre. They should be limited to two wards with four cots in each. Acute and infectious cases should not be admitted. A whole-time nurse should be in charge by day and one by night.

